# Titanate Nanotubes Decorated Graphene Oxide Nanocomposites: Preparation, Flame Retardancy, and Photodegradation

**DOI:** 10.1186/s11671-017-2211-9

**Published:** 2017-07-05

**Authors:** Bin Sang, Zhi-wei Li, Xiao-hong Li, Lai-gui Yu, Zhi-jun Zhang

**Affiliations:** 1 National & Local Joint Engineering Research Center for Applied Technology of Hybrid Nanomaterials, Henan University, Kaifeng, 475004 People’s Republic of China; 2 Collaborative Innovation Center of Nano Functional Materials and Applications of Henan Province, Henan University, Kaifeng, 475004 People’s Republic of China

**Keywords:** Titanate nanotube, Graphene oxide, Flame retardant, Photodegradation

## Abstract

Most polymers exhibit high flammability and poor degradability, which restrict their applications and causes serious environmental problem like “white pollution.” Thus, titanate nanotubes (TNTs) were adopted to decorate graphene oxide (GO) by a facile solution method to afford TNTs/GO nanocomposites with potential in improving the flame retardancy and photodegradability of flexible polyvinyl chloride (PVC). Results show that the as-prepared TNTs/GO can effectively improve the thermal stability and flame retardancy than TNTs and GO, especially, the peak heat release rate and total heat release were reduced by 20 and 29% with only 2.5 wt.% loading. And more, the TNTs/GO also improve the photodegradability of PVC compared with the neat PVC. The reasons can be attributed to synergistic flame-retardant and photocatalytic effects between TNTs and GO. The present research could contribute to paving a feasible pathway to constructing polymer-matrix composites with desired flame retardancy and photodegradability, thereby adding to the elimination of white pollution caused by polymers.

**Electronic supplementary material:**

The online version of this article (doi:10.1186/s11671-017-2211-9) contains supplementary material, which is available to authorized users.

## Background

Polymer-based materials are widely used in our daily lives and many industrial fields, due to their good properties such as low weight to strength ratio, relatively low cost, and good physical and chemical stability. However, most polymers are flammable and could cause potential hazard to human’s life and property, owing to their organic nature [1–4]. In the meantime, they usually exhibit chemical inertness and non-biodegradability, thereby producing severe white pollution to contaminate soil and water [5–8]. To deal with these issues, many researchers have made efforts to construct novel flame retardants in order to improve the flame retardancy and reduce the waste pollution of polymers.

For overcoming the flammability of polymer, researchers have explored a variety of strategies in the past decades [9]. It has been found that the introduction of nano-fillers is effective in improving the flame retardancy of polymer matrix, and non-toxic and environmentally friendly flame-retardant additives are of special significance in responding to people’s environmental concern. Among a variety of non-toxic and environmentally friendly additives, graphene-based materials are potentially attractive, because graphene and graphene oxide (GO) with layered structure and high specific surface area can act as barriers to inhibit heat release and prevent combustion gases from contact with flame [10–12]. Particularly, graphene or GO as a significant adjuvant can be combined with inorganic nanomaterials to afford promising candidates of flame retardants [13–15]. This is ascribed to the fact that the combination of two or more components can often present a synergism or integrate different flame retarding models, thereby offering an unexpected enhancement in the properties of composites. For example, inorganic nano-fillers, metals or metal derivative-based nanomaterials, such as Ce-MnO_2_ [16], TiO_2_ [17, 18], MoS_2_ [19], layered double hydroxide [20], and ZnSn(OH)_6_ [21] can be readily combined with graphene to provide graphene-based flame retardants.

The abovementioned synergistic strategy makes sense in improving the flame retardancy of polymer. However, it would still be infeasible in engineering unless the white pollution of polymer is simultaneously reduced or even eliminated. Currently available routes to dealing with the white pollution of polymer cover landfill and incineration. Landfill and incineration, nevertheless, can often cause a serious secondary pollution, such as contamination of soil and water by landfill as well as the release of toxic gas during incineration. This bottleneck, fortunately, could be overcome by applying sunlight to photodegrade waste polymer in an efficient and environmentally acceptable mode [5]. For example, TiO_2_, an important solid-phase photocatalyst, can be incorporated in polystyrene to afford polystyrene-TiO_2_ nanocomposite film that can be efficiently photocatalytically degraded under ultraviolet (UV) illumination in air [22, 23]. Vitamin C (VC)-modified TiO_2_ can endow photodegradable polystyrene-TiO_2_ nanocomposite films with a high photodegradation efficiency, which is attributed to the formation of a Ti^IV^–VC charge-transfer complex with five-member chelate ring structure that can prolong the separation of rapidly photogenerated charge [24].

In the present research, therefore, we try to combine proper flame-retardant additive with phtodegradation additive in order to simultaneously improve the flame retardancy and photodegradability of flexible polyvinyl chloride (PVC), a thermoplastic widely used in the fields of electronic industry, household electrical appliances, and building materials. We pay special attention to one dimensional titanate nanotubes (TNTs) rather than titania nanoparticles with a relatively small specific surface area, because TNTs combined with GO could have desired flame-retardant properties and photocatalytic activity towards polymer [17, 24]. Such a combination strategy might be feasible, because TNTs could catalyze charring and form a net-work structure which acts as an effective barrier to resist the release of flammable gases and change degradation pathway [25, 26]. In the meantime, TNTs with radical adsorption effect exhibit excellent smoke suppression ability as well as excellent photocatalytic activity towards Rhodamine B or waste water treatment. This article reports the preparation of TNTs decorated graphene oxide nanocomposites (TNTs/GO) by a facile solution reaction route. It also deals with the flame retardancy and photodegradation of TNTs/GO-PVC composites, with the emphasis being placed on the strategy to simultaneously improve the flame retardancy and reduce the white pollution of polymer.

## Methods

### Materials

PVC (for injection molding) was purchased from Tianjin Botian Chemical Company Limited (Tianjin, China). Commercial sodium titanate nanotubes (NaTA) were supplied by Engineering Technology Research Center for Nanomaterials (Jiyuan, China). Graphite powder (spectrally pure) was purchased from Sinopharm Chemical Reagent Company Limited (Shanghai, China). Ethanol (C_2_H_5_OH) was purchased from Anhui Ante Food Company Limited (Suzhou, China). Reagent grade concentrated sulfuric acid (98%), 30% H_2_O_2_ solution, hydrochloric acid, and 1, 2-ethanediamine (C_2_H_4_(NH_2_)_2_) were provided by Tianjin Kermel Chemical Reagent Company (Tianjin, China). Deionized water was prepared at our laboratory. All reagents were used as received without further purification.

### Preparation of GO Nanosheets and TNTs/GO Nano-filler

GO nanosheets were prepared from purified natural graphite through the method reported by Hummers and Offeman [27, 28]. TNTs/GO nano-fillers were prepared by a simple and practical solution method. In a typical procedure, 1.5 g of NaTA was added to 150 mL of H_2_O under mild stirring with the assistance of sonication, and the pH of the solution was adjusted to 1.6 with hydrochloric acid. After 30 min of stirring, 0.1 g of the as-prepared GO was added to the solution and sonicated for 1 h to afford a uniform suspension. The suspension was transferred into a 250-mL flask and maintained at 70 °C for 5 h. Upon completion of reaction, the precipitate was collected by filtration and washed several times with distilled water and ethyl alcohol to remove remnant impurities. The as-obtained precipitate was dried at 60 °C for 18 h to provide the TNTs/GO nano-filler.

### Preparation of TNTs/GO-PVC Composites

TNTs/GO-PVC composites filled with different contents of TNTs/GO nano-fillers were prepared with the method reported in our previous research [29]. A series of PVC composites denoted as PVC 0.5, PVC 1.5, PVC 2.5, and PVC 3.5 (mass fraction; the same hereafter except for explanation) were prepared in the same manners except that different dosages of TNTs/GO were incorporated. In addition, PVC composites with 2.5% of TNTs and GO (TNTs-PVC and GO-PVC) were also prepared under the same condition for comparative studies.

### Preparation of TNTs/GO-PVC Film

PVC powder (39 g) was suspended in 30 mL of tetrahydrofuran under 2 h of ultrasonic vibration; then, TNTs/GO (1 g) was dissolved in the suspension under 24 h of continuous vigorous stirring. Upon completion of stirring, the mixture was spread on a glass plate and dried for 72 h in an airtight vacuum vessel to afford the TNTs/GO-PVC film.

### Characterization

X-ray powder diffraction (XRD) patterns were collected with an X′ Pert Pro diffractometer (Cu *K*α radiation; *λ* = 0.15418 nm, operation voltage 40 kV, current 40 mA). A JEM-2010 transmission electron microscope (TEM) was performed to observe the morphology and microstructure of various products. X-ray photoelectron spectroscope (XPS) analysis was performed on an Axis Ultra multifunctional X-ray photoelectron spectrometer, using Al Kα excitation radiation (hv = 1486.6 eV). Raman spectra were recorder on a Renishaw inVia spectrometer, laser excitation light at 532 nm. Thermogravimetric analysis (TGA) and differential thermal analysis (DTA) were conducted with a DSC6200 thermal analyzer at the scanning rate of 10 °C/min. A JF-3 oxygen index meter was employed to measure the LOI values of the specimens with dimensions of 100 × 6.5 × 3 mm^3^. A WDW-10D microcomputer control electronic universal testing machine (Jinan Test Machine Company Limited; Jinan, China) was performed to determine the tensile strength of PVC-matrix composites. Cone calorimeter (Fire Testing Technology, UK) tests were conducted following the procedures described in ISO5660. Each specimen with the dimensions of 100 × 100 × 3 mm^3^ was exposed to 35 kW/m^2^ heat flux. The dispersion state of the additives in PVC matrix and the topography of residue chars were observed with a Nova Nano SEM 450 scanning electron microscope (SEM). An UV accelerated weathering tester (UV-II, Shanghai Pushen Chemical Machinery Co. Ltd.; Shanghai, China) was run to evaluate the photocatalytic degradation behavior of the rectangular PVC-matrix nanocomposite film (size 7.5 cm × 15 cm) under 45 °C and 40% humidity.

## Results and Discussion

### Microstructure of TNTs/GO Nano-filler

**Fig. 1:**
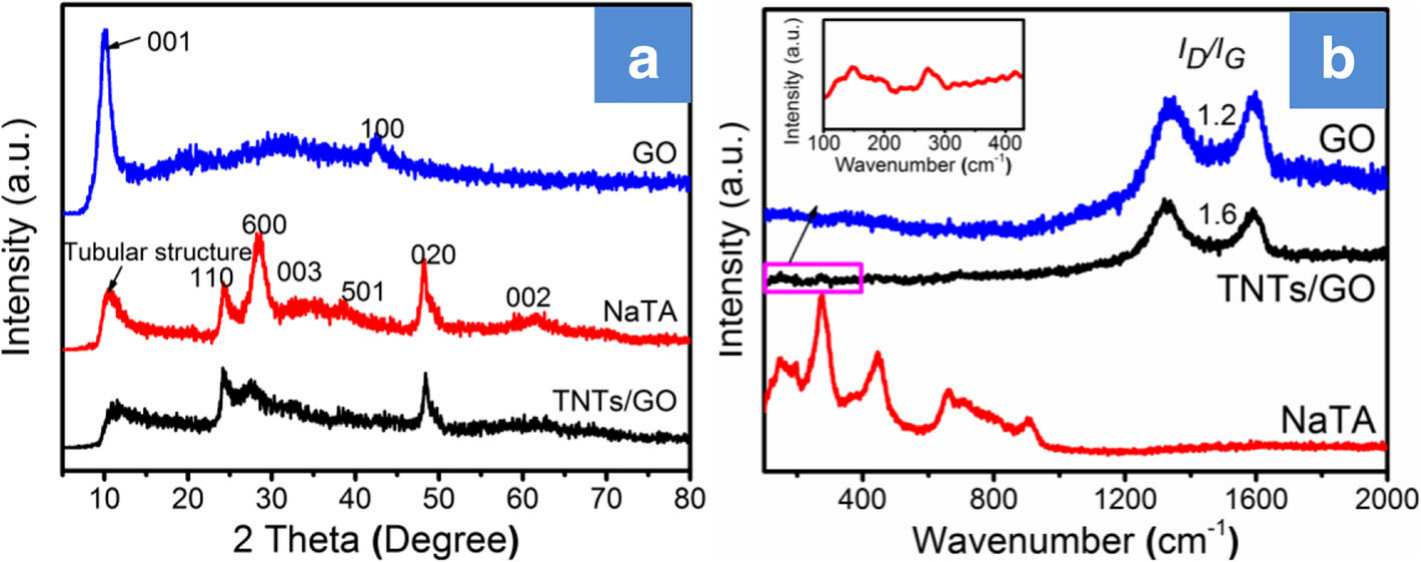
XRD patterns **a** and Raman patterns **b** of GO, NaTA, and TNTs/GO nanocomposites. The XRD patterns displayed that GO was exfoliated into sheets. The Raman patterns further verified the attendance of GO and proved that TNTs are successfully incorporated into GO nanosheets to provide TNTs/GO nanocomposite

Figure 1a shows the XRD patterns of GO, NaTA, and TNTs/GO nano-filler. GO has a major (001) diffraction peak at 2*θ* = 10.3°, and it corresponds to an interplanar spacing of 0.84 nm [30]. This means that graphite has been oxidized and completely exfoliated into sheets. The diffraction peaks of NaTA can be well indexed to Na_2 − *x*_H_*x*_Ti_2_O_5_·H_2_O, as reported elsewhere [31]. The diffraction patterns of TNTs/GO nanocomposite are similar to those of NaTA. However, the peak intensity of the TNTs/GO nanocomposite at 28° is lower than that of NaTA, which could be ascribed to the gradual transformation in the crystalline structure of NaTA yielding TNTs during the fabrication of the nanocomposite [32]. Namely, the replacement of Na^+^ with H^+^ leads to the decrease in the Na:H ratio of the titanate in this progress [31]. Moreover, the TNTs/GO nanocomposites exhibit no signals of any other phase of GO, which is possibly because TNTs are inserted into the GO layers to cause enhanced exfoliation of GO [33]. These XRD data demonstrate that the attachment of TNTs to the GO nanosheets contributes to preventing the aggregation and restacking of the as-synthesized GO.

Raman scattering spectra were recorded to investigate the changes in the structure of the as-prepared carbonaceous materials. Figure 1b shows the Raman spectra of GO, NaTA, and TNTs/GO nanocomposite. GO and TNTs/GO nanocomposite exhibit two typical peaks of GO at about 1588 cm^−1^ (G band; derived from the in-plane vibration of sp^2^-bonded carbon atoms) and a peak at 1338 cm^−1^ (D band; associated with the vibrations of carbon atoms with sp^3^ electronic configuration of disordered graphene.) [34]. Besides, the peak intensity ratio of the D band to G band (*I*_D_/*I*_G_) is 1.2 for GO but 1.6 for TNTs/GO nanocomposite, which also proves that TNTs are successfully incorporated into GO nanosheets to provide TNTs/GO nanocomposite through the formation of Ti–C and Ti–O–C bonds and the reduction of GO, and they are will observed in FTIR data (see Additional file 1: Figure S1) [35, 36]. Moreover, aside from the predominant Raman peaks of GO, the TNTs/GO nanocomposite shows the characteristic peaks of NaTA, and these further indicates that TNTs have been successfully incorporated into GO nanosheets, which well conforms to relevant XRD.

**Fig. 2:**
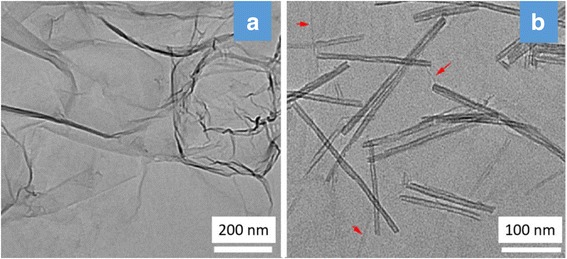
TEM images of **a** GO and **b** TNTs/GO nanocomposites. GO nanosheets exhibit a layered structure and a typical crumpled morphology, while the surface of TNTs/GO nanocomposite is relatively smooth and contains a small amount of unconspicuous and slight wrinkle, implying that the incorporation of TNTs can well prevent the aggregation and restacking of the GO nanosheets

Figure 2 shows the TEM morphology and microstructure of GO and TNTs/GO nanocomposite. GO nanosheets exhibit a layered structure and a typical crumpled morphology (Fig. 2a), due to their high specific area and surface energy. And the GO sheets are about one–two layers stack (as displayed in Additional file 1: Figure S2). The surface of TNTs/GO nanocomposite, however, is relatively smooth and contains a small amount of unconspicuous and slight wrinkle, and the aggregation and restacking of GO seem to be effectively prevented as compared with neat GO (Fig. 2b). This implies that the incorporation of TNTs can well prevent the aggregation and restacking of the GO nanosheets.

**Fig. 3:**
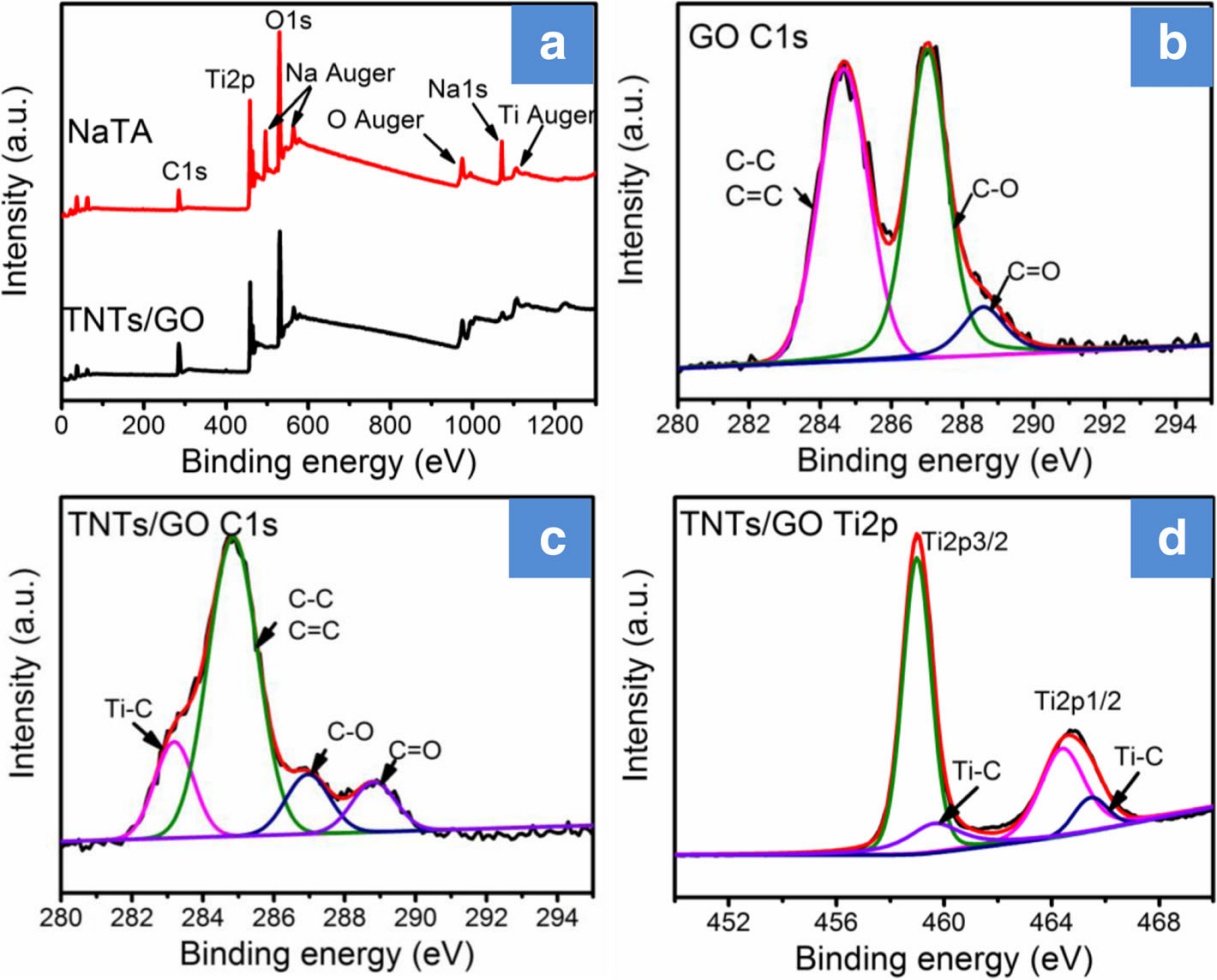
**a**–**d** XPS spectra of GO and TNTs/GO nanocomposites. Ti–C bonds are formed between GO and TNTs to provide TNTs/GO nanocomposite through a stable chemical attachment rather than a physical absorption

In order to further elucidate the interaction between GO and TNTs in TNTs/GO nanocomposites, we performed XPS measurements. As shown in Fig. 3, the C1s XPS spectrum of GO is fitted into three peaks attributed to *sp* ^2^-bonded carbons (C–C, C=C, 284.7 eV), epoxyl/hydroxyl (C–O, 286.9 eV), and carboxyl (C(O)O, 288.5 eV), respectively [37]. The C1s peak at 284.7 eV proves the attendance of 2D carbon structure, and the C1s peaks at 286.9 eV and 288.5 eV indicate a high percentage of oxygen-containing functional groups. It can be observed that the oxygen-containing groups of GO and TNTs/GO have changed during the synthetic process. In order to quantitatively confirm and compare the change of the oxygen-containing groups, we calculated the peak area ratios of oxygen-containing groups to the total carbon bonds. As listed in Table 1 (notes: *A* = *A*_CC_ + *A*_CO_ + *A*_C(O)_ + *A*_TiC_), the percentage of C–O bond of TNTs/GO is remarkably lower than that of GO, and the percentage of *sp* ^2^-bonded carbon increases from 46.25% of GO to 63.13% of the TNTs/GO nanocomposite. This indicates that GO is partly reduced and most of the oxygen-containing groups are removed from GO during the formation of TNTs/GO nanocomposite, which is also supported by relevant Raman data (the *I*_D_/*I*_G_ ratio of GO is smaller than that of the TNTs/GO nanocomposite). The reason might lie in that TNTs can reduce GO into graphene under UV or visible light photocatalytic process [38, 39]. Moreover, TNTs/GO nanocomposite shows a weak C1s peak at 283.2 eV, and this peak is assigned to Ti–C bond (corresponding Ti(2p3/2) and Ti(2p1/3) peaks emerge at 460.4 and 465.9 eV) [36].

**Table 1:** Peak area (*A*) ratios of the oxygen-containing bonds to the total carbon bonds (obtained by XPS)

Sample	Peak area ratio			
	*A*_CC_ */A*	*A*_CO_ */A*	*A*_C(O)_ */A*	*A*_TiC_ */A*
GO	0.4625	0.4527	0.0848	–
TNTs/GO	0.6313	0.1114	0.0931	0.1594

The possible formation process of Ti–C bonds can be described as following. TNTs have a scroll-type nanotube structure, and their (100) facets are of a stepped surface structure consisting of Ti and exposed O atoms [40]. In an acidic solution of pH = 1.6, the walls of TNTs will undergo dehydration and structure transformation to afford defects [32, 41]. As a result, Ti–C bonds are formed between GO and TNTs to provide TNTs/GO nanocomposite through a stable chemical attachment rather than a physical absorption. Since Ti–C bonds can facilitate the interfacial charge transfer between TiO_2_ and graphene [42], the high proportion of Ti–C bonds could be of special significance for the application of TNTs/GO nanocomposite in the photodegradation catalysis.

### Thermal Stability and Mechanical Properties of Flexible PVC Composites

**Fig. 4:**
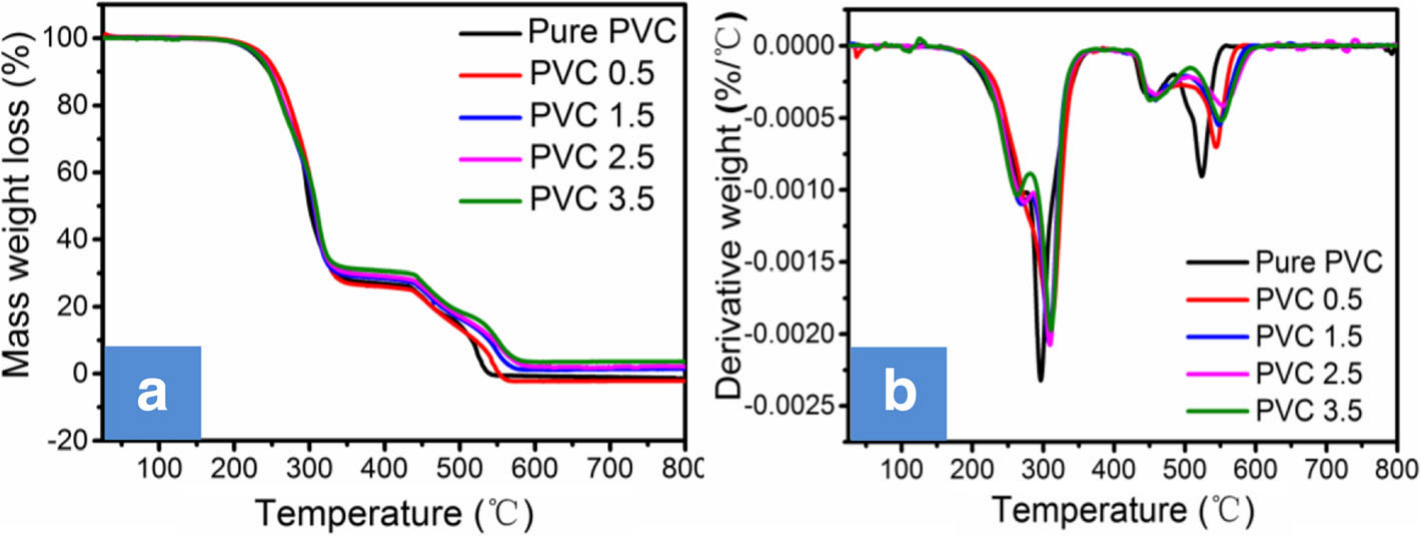
TGA **a** and DTG **b** curves of flexible PVC and PVC-matrix composites in air atmosphere. The thermal stability of the PVC-matrix nanocomposite was improved, which could be ascribed to the synergistic effects between TNTs and GO

The TGA and DTG curves of PVC and PVC-matrix composites filled with various contents of TNTs/GO are displayed in Fig. 4. Corresponding thermogravimetric data are summarized in Table 2, where the temperatures at which 5% (*T*_5%_), 50% (*T*_50%_), and maximum (*T*_max_) mass loss occur are described as the initial degradation temperature, half degradation temperature, and maximum degradation temperature, respectively. It can be seen that the *T*_5%_, *T*_50%_, and *T*_max_ of TNTs/GO-filled PVC composites are a little bit higher than those of pure PVC; and in particular, the PVC-matrix nanocomposite with 2.5% of TNTs/GO nano-filer has a quite higher *T*_max_ than the virgin PVC. These data indicate that TNTs/GO nano-filler can enhance the thermal stability of PVC. This could be attributed to the good dispersion state of TNTs/GO in PVC matrix (see Additional file 1: Figure S3), the good interfacial interaction between TNTs/GO and PVC molecules, and the synergistic effects between TNTs and GO. Namely, the good dispersion of TNTs/GO can promote the crosslinking of PVC chains; the GO nanosheets can act as physical barriers to inhibit the transport of heat and mass under the help of TNTs; the TNTs can catalyze charring and anchor in the char to enhance the stability of residue char. As a result, the underlying PVC matrix is protected, and the thermal stability of the PVC-matrix nanocomposite is improved.

**Table 2:** Thermogravimetric data of flexible PVC and PVC-matrix composites in air atmosphere

Sample	*T*_5%_/°C	*T*_50%_/°C	*T*_max1_/°C	*T*_max2_/°C
PVC 0	228	300	296	524
PVC 0.5	239	308	310	544
PVC 1.5	230	306	308	548
PVC 2.5	232	308	309	554
PVC 3.5	230	309	311	551

**Fig. 5:**
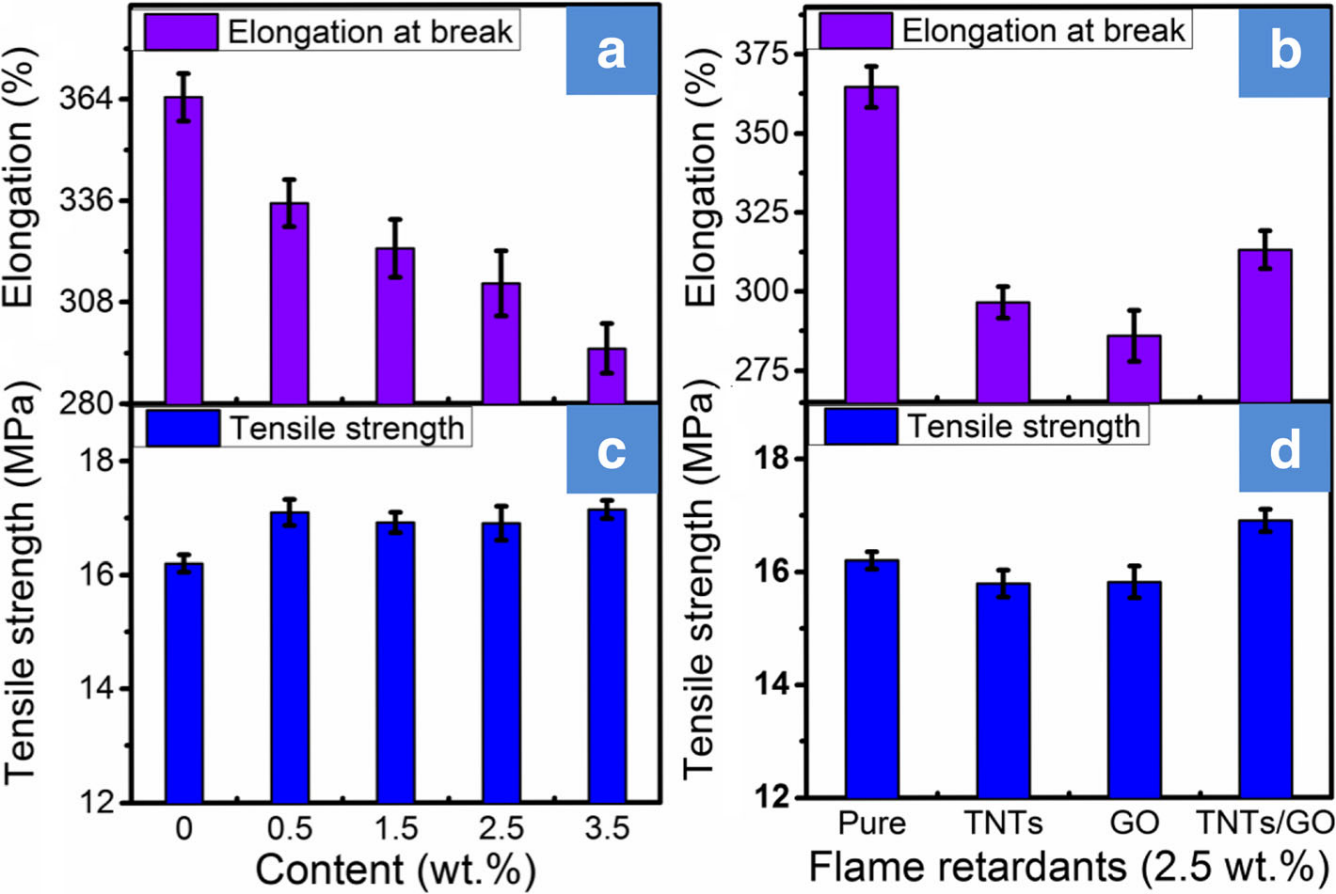
Tensile strength and elongation at break of flexible PVC and PVC-matrix composites with various content of TNTs/GO (**a**, **c**) and with 2.5 wt.% of GO or of TNTs and TNTs/GO (**b**, **d**). The incorporated TNTs/GO to PVC could enhance the tensile strength of the PVC-matrix composites, because the TNTs/GO nano-filler exhibits good exfoliation and dispersion that enhance the interfacial adhesion with the PVC matrix

The tensile strength at break of pure PVC and its composites with different contents of TNTs/GO nano-filler is presented in Fig. 5a, and that of the PVC filled with TNTs alone or GO alone is presented in Fig. 5b for a comparison. It can be seen that the incorporation of TNTs or GO causes a decrease in the tensile strength of PVC matrix, which is because the inorganic fillers exhibit poor compatibility and weak interaction with the PVC matrix. To our surprise, although the elongations at break of the PVC-matrix composites tends to decrease with increasing content of TNTs/GO nano-filler, the tensile strength of the PVC-matrix composites is always higher than that of neat PVC whether the content of TNTs/GO nano-filler is high or low. This could be because the TNTs/GO nano-filler exhibits good exfoliation and dispersion as well as enhanced interfacial adhesion with the PVC matrix and can transfer stress effectively.

### Flame Retardancy of Flexible PVC Composites

**Fig. 6:**
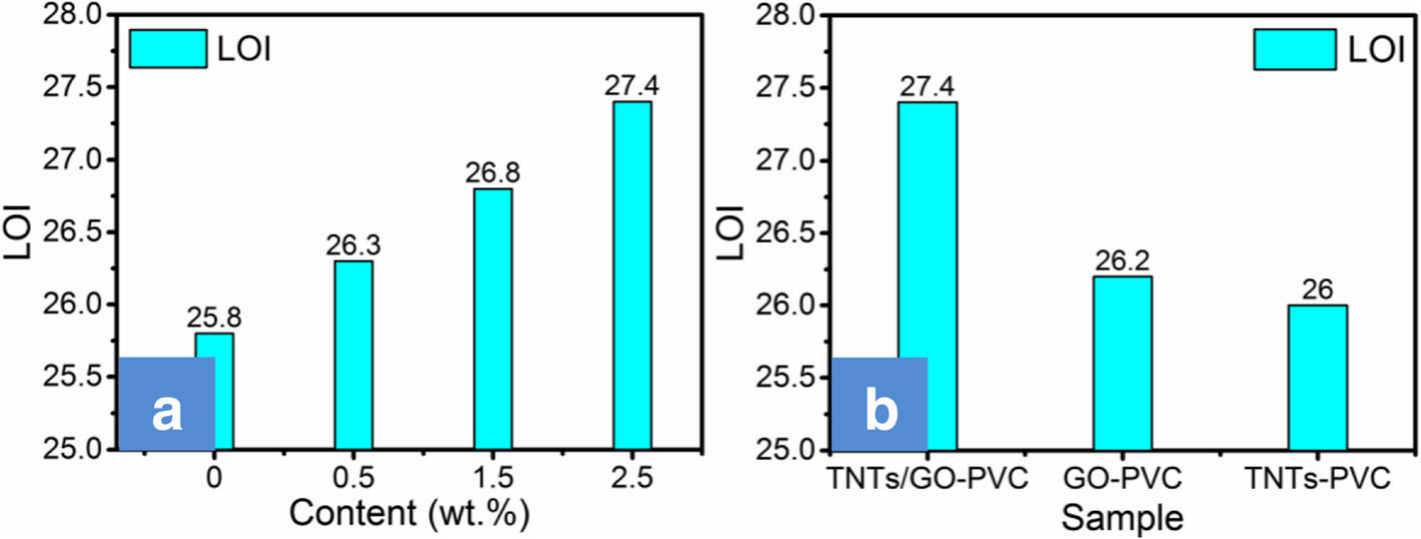
LOI values of flexible PVC and PVC-matrix composites with various content of TNTs/GO (**a**) and with 2.5 wt.% of GO or of TNTs and TNTs/GO (**b**). The PVC-matrix nanocomposites containing 2.5% of TNTs/GO nano-filler exhibits a maximum LOI of 27.4, higher than that of PVC filled with GO alone or TNTs alone. This demonstrates that there is the synergistic flame-retardant effect between TNTs and GO

Limiting oxygen index (LOI) is a criterion to screen inflammable materials. The LOI data of PVC and PVC-matrix composites are displayed in Fig. 6. The LOI value of the neat PVC is 25.8, corresponding to its inherent flammability. The LOI values of PVC filled with GO alone or TNTs alone are 26.2 and 26.0, respectively, which indicates that GO and TNTs can separately improve the flame retardancy of PVC to some extent. This is because GO exhibits a barrier effect, while TNTs can catalyze the formation of char and exhibits adsorption effect and radical adsorption effect. As to TNTs/GO-filled PVC composites, their LOI values tend to increase with increasing content of the nano-filler up to a mass fraction of 2.5%. Particularly, the PVC-matrix nanocomposites containing 2.5% of TNTs/GO nano-filler exhibits a maximum LOI of 27.4, higher than that of the PVC filled with GO alone or TNTs alone. This demonstrates that there is some kind of synergistic flame-retardant effect between TNTs and GO.

**Fig. 7:**
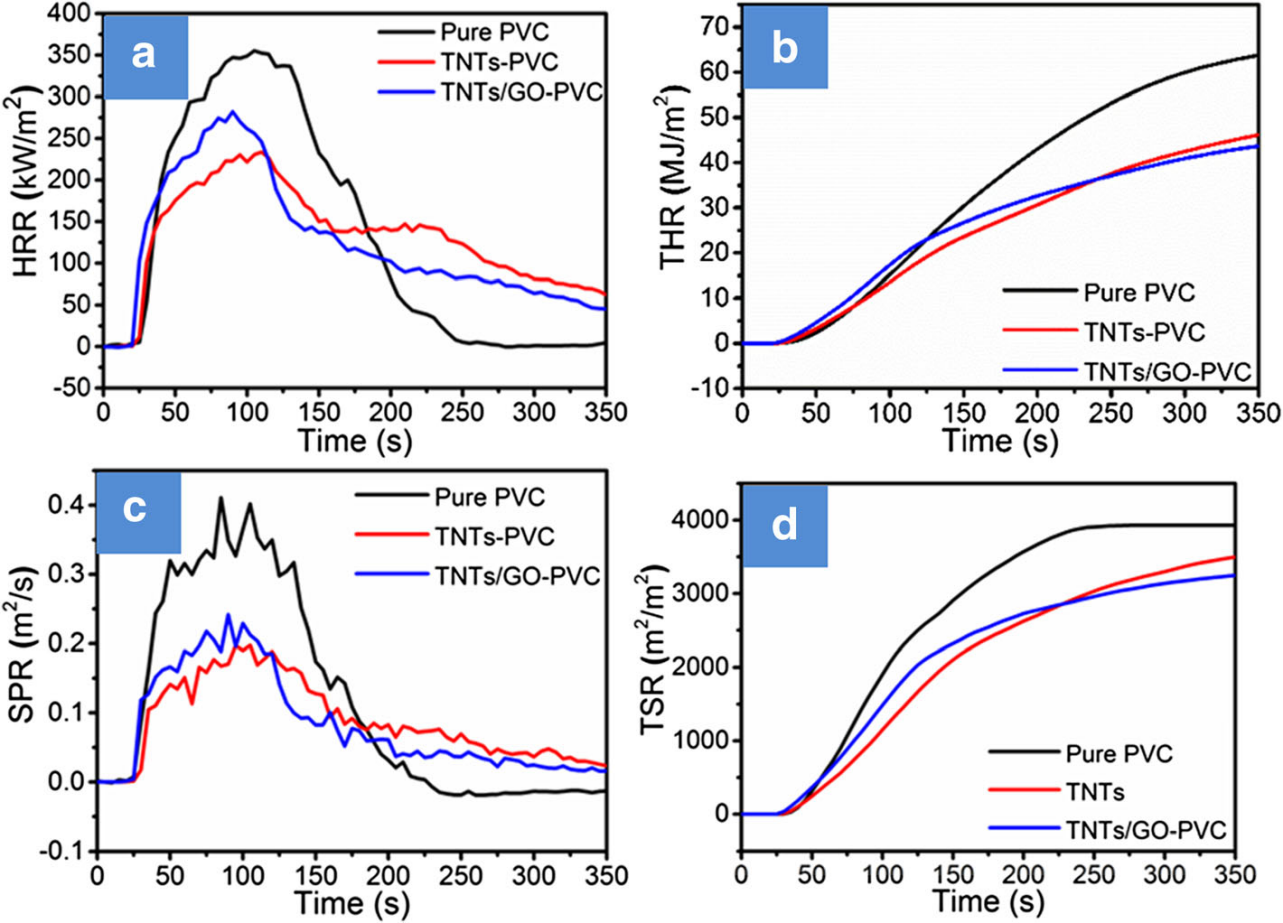
HRR **a**, THR **b**, SPR **c**, and TSR **d** versus time curves of pure flexible PVC and PVC-matrix composites obtained from cone calorimetry test at 35 kW m^−2^. The HRR curve of TNTs-filled PVC contains a second peak around 230 s, which means that the char is unstable and can be destroyed easily. The TNTs/GO inhibited the second heat release peak, and with 2.5% content, the pHRR value is 282.4 kW m^−2^, a reduction by 20.5% in comparison with that of neat PVC. This indicates that the char on the surface of the TNTs/GO-filled PVC composites is very stable and can act as a physical barrier to hinder the transmission of heat

Peak heat release rate (pHRR), total heat release (THR), total smoke release (TSR), and average specific mass rate (AMLR) are important parameters to evaluate the flammability of various materials under real-world fire conditions, and they can be obtained from cone calorimetry tests. Corresponding test results are shown in Fig. 7, and the data are summarized in Table 3. It can be seen that neat PVC has a sharp HRR peak with a pHRR value of 355.4 kW m^−2^ (Fig. 7a). Incorporating 2.5% TNTs into PVC drops the pHRR value down to 233.7 kW m^−2^, and such a reduction by 34.2% is attributed to the fast charring on the surface of filled PVC under the catalytic action of TNTs. However, the HRR curve of TNTs-filled PVC contains a second peak around 230 s, which means that the char is unstable and can be destroyed easily. In contrast, the PVC nanocomposite filled with 2.5% of TNTs/GO has a pHRR value of 282.4 kW m^−2^, a reduction by 20.5% in comparison with that of neat PVC. Moreover, the pHRR of TNTs/GO-filled PVC nanocomposite declines rapidly to a low value at 130 s and maintains steady with extending duration. This indicates that the char on the surface of the TNTs/GO-filled PVC composites is very stable and can act as a physical barrier to hinder the transmission of heat, thereby leading to greatly lowered THR, TSR, and AMLR.

**Table 3:** Cone calorimetric data of pure PVC and its composites

Sample	pHRR (kW m^−2^)	THR (MJ m^−2^)	TSR (m^2^ m^−2^)	AMLR (g s^−1^)
Pure PVC	355.4	65.3	3936.8	20.4
TNTs-PVC	233.7	51.9	3670.9	14.6
TNTs/GO-PVC	282.4	46.3	3322.7	14.2

**Fig. 8:**
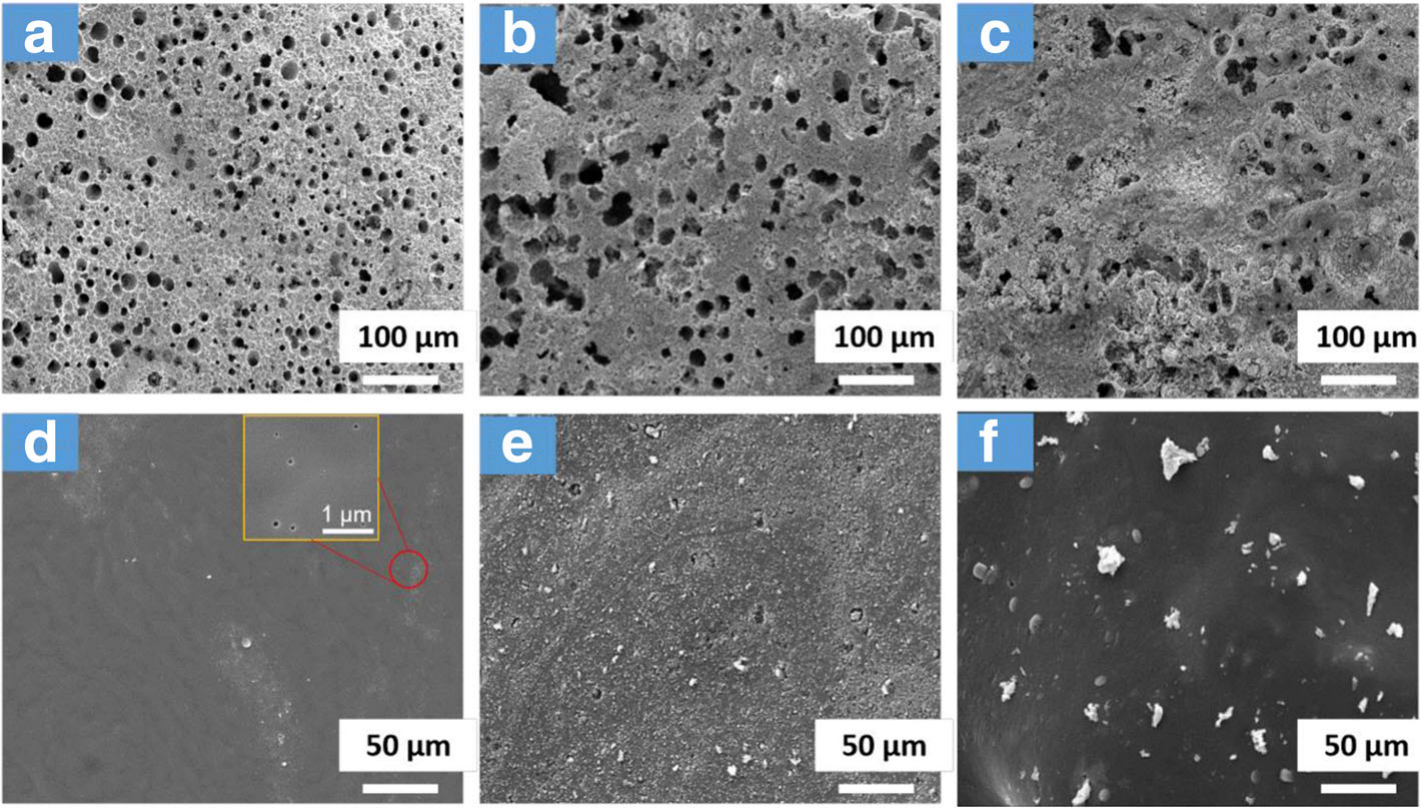
SEM images of exterior and interior morphology of the residue chars of PVC (**a**, **d**) TNTs-PVC (**b**, **e**) and TNTs/GO-PVC (**c**, **f**) composites. The char of TNTs/GO-PVC was more compact and continuous than that of the neat PVC or TNTs-PVC

To further confirm the flame-retardant mechanism of PVC-matrix composites, we conducted SEM analyses of the residual chars. As shown in Fig. 8a–c, all of the exterior chars have holes with different sizes and distributions. This means that the holes on pure PVC occupy a large area and are deep enough to penetrate the bulk. The exterior chars of TNTs-PVC are similar to those of neat PVC, but the holes of the former are much bigger. These holes can act as transport channels for heat and mass. Therefore, neat PVC and TNTs-PVC allow the heat to easily transfer from combustion surface to polymeric matrix and flammable organic volatiles to escape from underlying matrix to combustion zone. On the contrary, the exterior chars of TNTs/GO-PVC contain fewer holes which are mostly impotent. This means that the channels for heat and mass transport are cut off. The interior chars of TNTs/GO-PVC (Fig. 8f) are compact and continuous at the surface and contain titanium dioxide anchored inside, while those of neat PVC and TNTs-PVC contain many small cracks or holes. This, in association with the much smaller ID/IG of TNTs/GO-PVC (1.1) as compared with that of neat PVC (1.6; see Additional file 1: Figure S4), suggests that TNTs/GO can transform carbon sources into char, thereby adding to the flame retardancy of the PVC-matrix composites. In one word, the stable, compact, and continuous char layers of TNTs/GO-PVC composites can act as good physical barriers to reduce the pHRR, THR, SPR, and TSR and resist thermal shock as well.

**Fig. 9:**
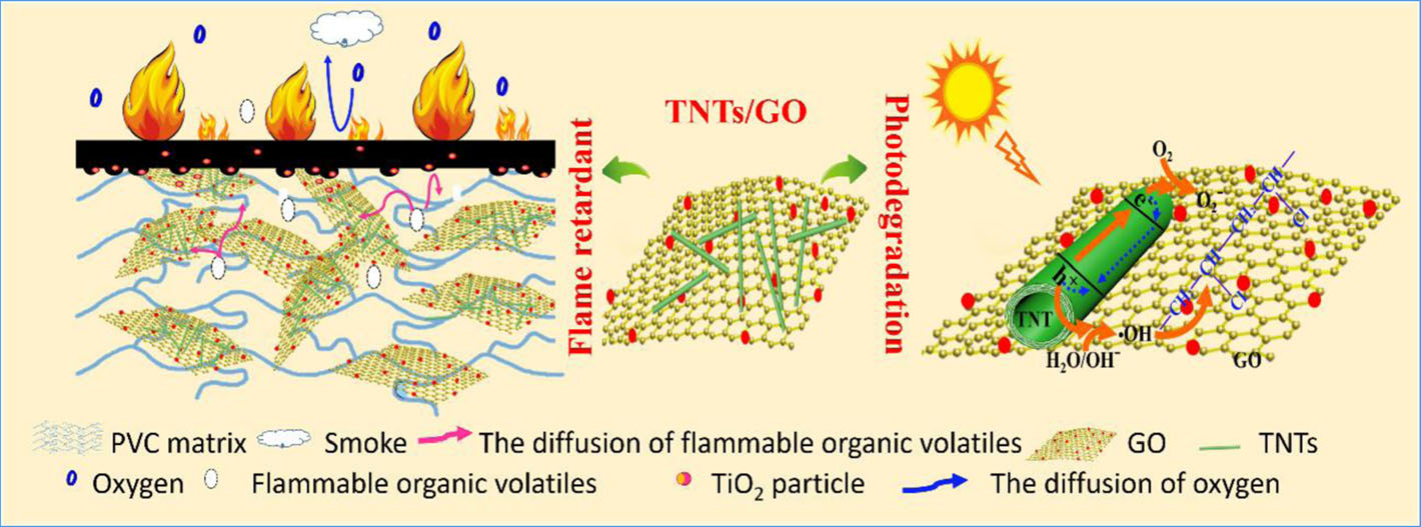
Schematic illustration of flame-retardant and photocatalytic degradation mechanism of TNTs/GO

The synergistic flame-retardant effect between TNTs and GO is schematically illustrated in Fig. 9. Firstly, TNTs decorated on the surface of GO nanosheets suppress the re-stack of GO and promote the uniform dispersion of TNTs/GO in the PVC matrix, thereby enhancing the thermal stability of PVC. Secondly, TNTs and GO with large surface areas make contributions to absorbing pyrolysis gas during combustion, prolonging the diffusion way of volatile gases, and increasing the catalyst residence time, thereby allowing easy transformation of the pyrolysis gases into carbonaceous char under the catalytic action of TNTs. Thirdly, the GO skeleton can act as a template for the carbonaceous char and promote the formation of multiple char under the help of TNTs, thereby affording a continuous and compact char layer. Finally, titanium dioxide transformed from TNTs during combustion (see Additional file 1: Figure S4 for more details) can be anchored in the char layers to enhance the thermal stability of the char layers, which makes it feasible for the char layers to more effectively resist the thermal shock at the second peak, prevent the heat, and reduce smoke release. These multiple factors jointly function and account for the enhanced flame retardancy of PVC-matrix composites.

### Photodegradation of TNTs/GO-filled PVC Film

**Fig. 10:**
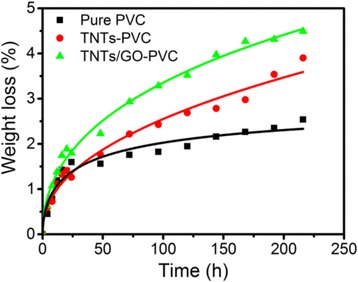
Weight loss of pure PVC and PVC-matrix nanocomposite films under UV-light irradiation (40% humidity). Incorporated TNTs/GO to PVC could accelerate the photo degradation of PVC. The large interfacial contact surface area and strong interaction between TNT and GO could help to reduce the charge resistance and the charge recombination rate, and the Ti–C bond could also promote the interfacial charge transfer, thereby accelerating the degradation of TNTs/GO-PVC nanocomposite films under UV irradiation

As mentioned previously, the TNTs/GO nano-filler could act as a photocatalyst for PVC degradation. Figure 10 presents the weight loss of TNTs-PVC film and TNTs/GO-PVC film under UV illumination. The weight loss of TNTs/GO-PVC film decreases gradually with extending irradiation time and undergoes a reduction of 4.6% in 216 h, while the PVC film undergoes only 2.5% of weight loss under identical experimental condition. Besides, the weight loss rate of TNTs/GO-PVC film is higher than those of the PVC film and TNTs-PVC film. This could be attributed to two aspects. On the one hand, reduced graphene oxide nanosheets have a large surface area, providing a large interfacial contact surface area and strong interaction with TNT that helps to reduce the charge resistance and the charge recombination rate, which makes it feasible for reduced graphene oxide nanosheets to act as excellent electron acceptors and well transport charge [43–46]. On the other hand, the Ti–C bond can also promote the interfacial charge transfer, thereby accelerating the degradation of TNTs/GO-PVC nanocomposite films under UV irradiation [42]. The catalytic efficiency of the TNTs/GO nano-filler, however, is fairly low. This is possibly because the incompletely reduced GO nanosheets have a large amount of oxygen-containing functional groups and defects that hinder the charge transport while the dehydration of TNTs at pH = 1.6 also causes defects in the nanotubes to facilitate charge recombination. Therefore, the oxygen-containing functional groups on the surface of graphene nanosheets are simultaneously beneficial to the flame resistance and the photodegradation of PVC-matrix composites.

**Fig. 11:**
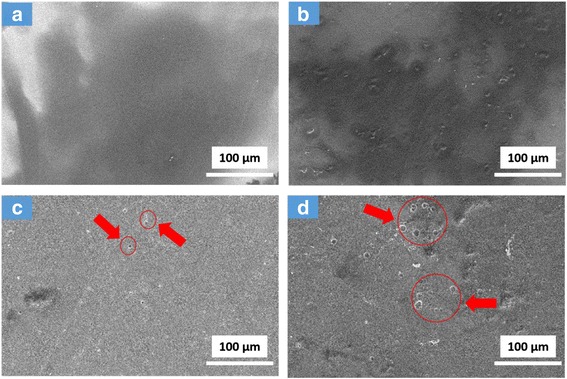
SEM images of the morphology of PVC (**a**, **c**) and TNTs/GO-PVC (**b**, **d**) films before and after 72 h of UV irradiation. More and large cavities were formed on the surface of TNTs/GO-PVC film after UV irradiation, which indicates that the TNTs/GO could promote the photocatalytic degradation of PVC under UV irradiation

Figure 11 shows the surface morphologies of PVC film and PVC-matrix nanocomposite film before and after 216 h of UV irradiation. It can be seen that the two kinds of PVC films are smooth before UV irradiation. After UV irradiation, many holes are formed on the surface of PVC film (Fig. 11c, d), which indicates that the film undergoes obvious decomposition under UV irradiation. In the meantime, more and large cavities are formed on the surface of TNTs/GO-PVC film after UV irradiation (Fig. 11d), which indicates that the TNTs/GO nano-filler does promote the photocatalytic degradation of PVC under UV irradiation.

**Fig. 12:**
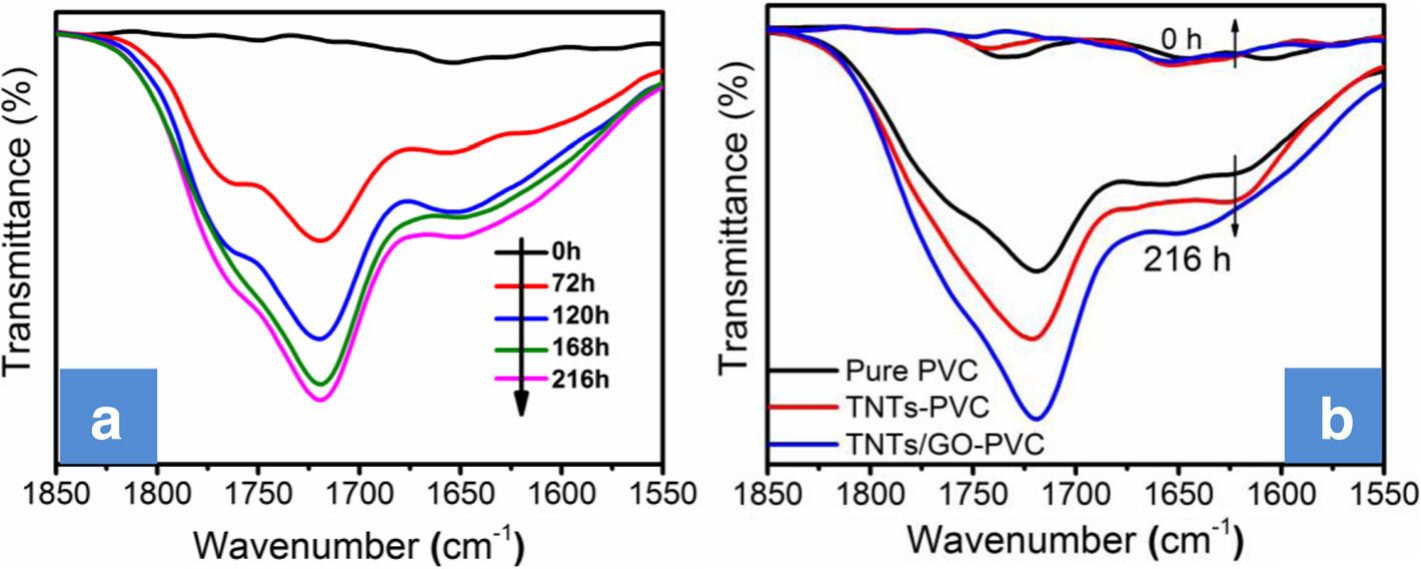
FTIR spectra of the carbonyl (C=O) groups of TNTs/GO-PVC film versus UV irradiation time (**a**) and before and after photodegradation (**b**). The intensity of the C=O absorption peak of TNTs/GO-PVC film increases continually with increasing irradiation time; it is more pronounced than that for neat PVC film and for TNTs-PVC film. This further demonstrates that the TNTs/GO nano-filler can indeed promote the photo-oxidation reaction of the PVC matrix

The functional groups in the PVC-matrix nanocomposite films were also monitored by FTIR analysis. As shown in Fig. 12a, the Raman peak of carbonyl (C=O) groups at 1680–1800 cm^−1^ proves that the TNTs/GO-PVC nanocomposite film does undergo degradation reaction during UV irradiation. Moreover, the intensity of the C=O absorption peak increases continually with increasing irradiation time; and the increase in the intensity of the C=O absorption peak is more pronounced for TNTs/GO-PVC film than for neat PVC film and for TNTs-PVC film (Fig. 12b). This further demonstrates that the TNTs/GO nano-filler can indeed promote the photo-oxidation reaction of the PVC matrix.

## Conclusions

In summary, TNTs/GO nanocomposites were prepared through a facile solution method. The as-prepared TNTs/GO nano-filler can simultaneously improve the flame retardancy and photodegradability of PVC, which could be attributed to the synergistic effects between TNTs and GO. On the one hand, TNTs can suppress the re-stack of GO and promote the uniform dispersion of TNTs/GO in the PVC matrix; GO nanosheets can act as electron acceptors to reduce the charge resistance and charge recombination rate, and the GO skeleton can also act as a template for the carbonaceous char and promote the formation of multiple char under the help of TNTs. On the other hand, titanium dioxide transformed from TNTs during combustion can be anchored in the char layers to enhance the thermal stability of the char layers and accelerate the photodegradation of PVC matrix under UV irradiation. As a result, TNTs/GO-PVC composites exhibit enhanced flame retardancy and photodegradability than TNTs-PVC and GO-PVC counterparts. The present research, hopefully, would help to provide a promising strategy for constructing polymer-matrix composites with simultaneously improved flame retardancy and photodegradability, thereby shedding light on dealing with the white pollution of commonly used polymers. Further researches are to be conducted concerning the enhancement in the flame-retardant and photodegradation efficiencies of the TNTs/GO nano-filler.

## Additional file

Additional file 1: Figure S1. AFM images of GO sheets, the thickness of the GO fragment is ca. 1.72 nm. **Figure S2.** FTIR spectra of GO, TNTs, and TNTs/GO nanocomposites. **Figure S3.** SEM images of the fracture surface of flexible PVC (a), and GO-PVC (b), TNTs-PVC (c), and TNTs/GO (d). The inset image of d is Ti-element EDA mapping of TNTs/GO composites. **Figure S4.** XRD patterns (a) and Raman patterns (b) of char of PVC and TNTs/GO-PVC.
