# Supplementary material for: Titanate Nanotubes Decorated Graphene Oxide Nanocomposites: Preparation, Flame Retardancy, and Photodegradation
Source: Nanoscale Res Lett. 2017 Jul 5;12:441. doi: 10.1186/s11671-017-2211-9 (PMC5498429; doi:10.1186/s11671-017-2211-9)
Supplement: Additional file 1: Figure S1. — AFM images of GO sheets, the thickness of the GO fragment is ca. 1.72 nm. Figure S2. FTIR spectra of GO, TNTs, and TNTs/GO nanocomposites. Figure S3. SEM images of the fracture surface of flexible PVC (a), and GO-PVC (b), TNTs-PVC (c), and TNTs/GO (d). The inset image of d is Ti-element EDA mapping of TNTs/GO composites. Figure S4. XRD patterns (a) and Raman patterns (b) of char of PVC and TNTs/GO-PVC. [file 11671_2017_2211_MOESM1_ESM.docx]

Titanate nanotubes decorated graphene oxide nanocomposites: preparation, flame retardancy and photodegradation

Bin Sang^1,2^, Zhi-wei Li^1,2,*^, Xiao-hong, Li^1,2,*^, Lai-gui Yu^1,2^ and Zhi-jun Zhang^1,2^

*^1^ National & Local Joint Engineering Research Center for Applied Technology of Hybrid Nanomaterials, Henan University, Kaifeng 475004, People’s Republic of China*

*^2^ Collaborative Innovation Center of Nano Functional Materials and Applications of Henan Province, Henan University, Kaifeng 475004, People’s Republic of China*

**Corresponding author. E-mail: zhiweili@henu.edu.cn; Fax: +86 371 25152066*


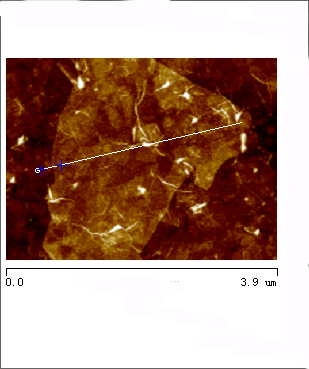

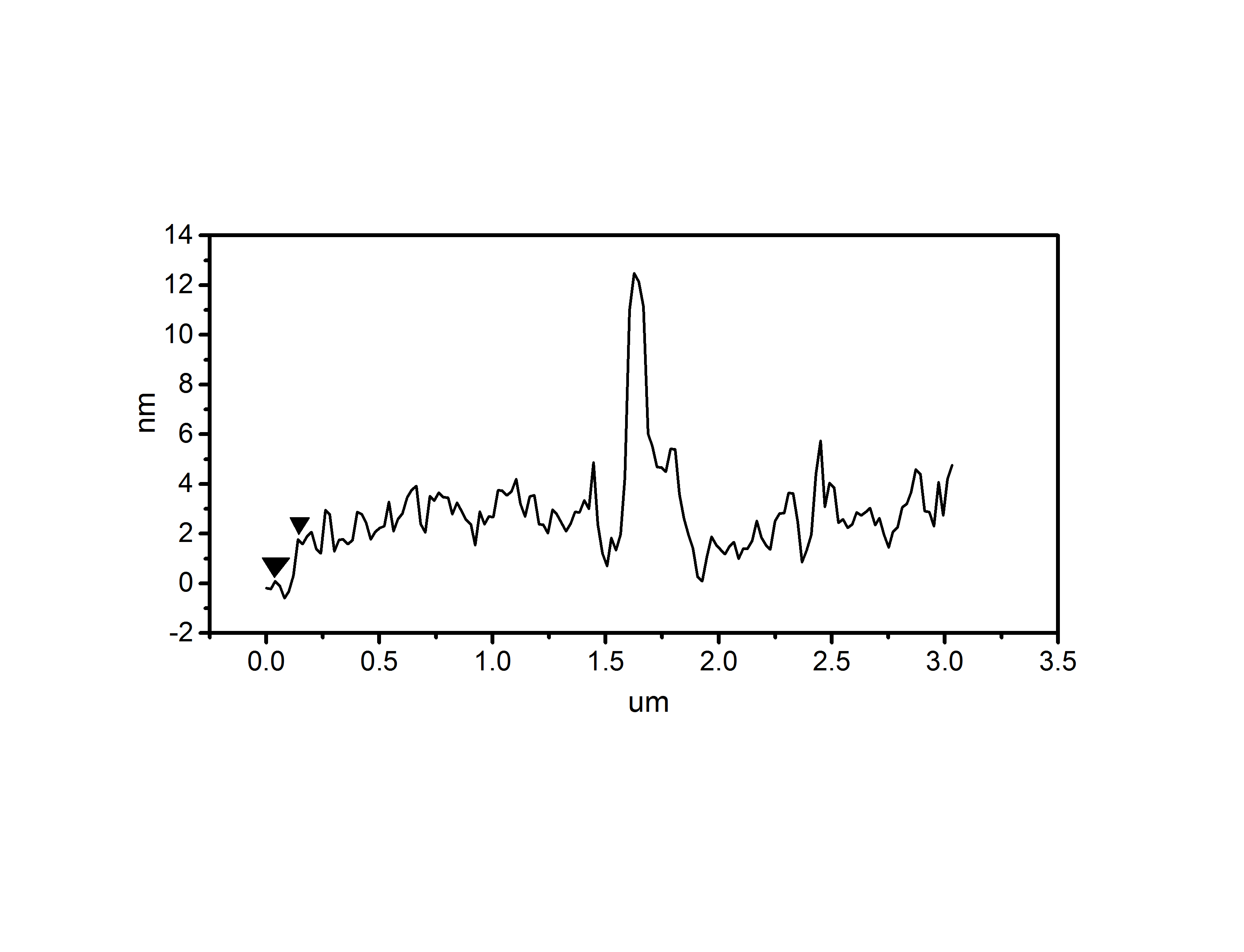


**Supplementary Figure S1.** AFM images of GO sheets, the thickness of the GO fragment is ca. 1.72nm.

The AFM images of GO sheets show a GO nanosheet with wrinkle-like features, which is respond to the TEM results, and the apparent thickness is ca. 1.72 nm, meaning that the GO was exfolited into one-two layers for precursor of TNTs/GO [1].

**Supplementary Figure S2.** FTIR spectra of GO, NaTA and TNTs/GO nanocomposites.

The FT-IR spectra of raw GO, NaTA, and TNTs/GO nanocomposite are shown in Supplementary Figure S2. The absorption at 1389 and 1066 cm^-1^ are assigned to the C-O stretching vibration of -COOH and C-OH groups situated at edges of GO nanosheets, which decreased in TNTs/GO, this could be ascribed to the TNTs can reduce GO into graphene in an UV or visible light photocatalytic process [2, 3]. More importantly, the broader absorption band around 500-100 cm^-1^ in the composite can be considered the combination of Ti-O-Ti and Ti-O-C vibrations, implying that GO was decorated with TNTs and in response to the XRD and Raman results.

**Supplementary Figure S3.** SEM images of the fracture surface of flexible PVC (a) and GO-PVC (b), TNTs-PVC (c), and TNTs/GO (d). The inset image of d is Ti-element EDA mapping of TNTs/GO composites.

It usually requires a strong interfacial adhesion between fillers and matrix and the homogeneous dispersion of the fillers in the matrix to acquire good mechanical properties, which also plays an important role in flame retardant properties [4, 5]. Supplementary Figure S2a shows that the fracture surface of pure PVC, and the relatively smooth fracture area corresponds to a typical brittle fracture feature. In contrast, the fracture surfaces of the filled PVC-matrix composites, b, c, and d, show rougher and blurred fractured surfaces with many lines and wrinkles, corresponding to typical tough fracture feature. And TNTs-PVC and GO-PVC exhibit obvious aggregation (marked in red tracks). On the contrary, TNTs/GO-PVC is nearly free of any significant aggregation, this indicates that TNTs/GO is well dispersed in the PVC matrix and there is a strong interfacial adhesion between TNTs/GO and PVC matrix. The element compositions in the selected areas (inset in Supplementary Figure S2d) were determined by EDX to survey the dispersion of TNTs/GO. It is observed that Ti element has a homogeneous distribution, which further suggests that TNTs/GO nano-filler exhibits good dispersion in polymer matrix.


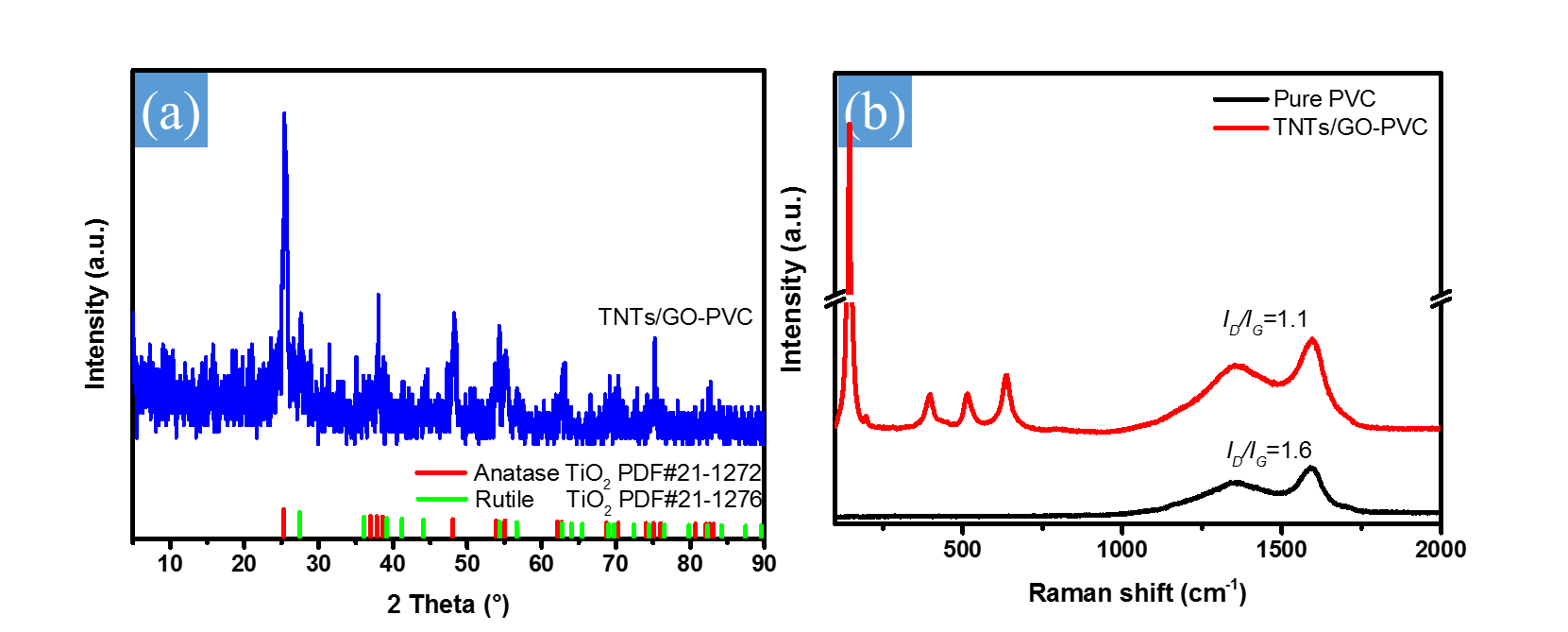


**Supplementary Figure S4.** XRD patterns (a) and Raman patterns (b) of char of PVC and TNTs/GO-PVC.

XRD and Raman were performed to verify the components of the char residues (supplementary Figure S3). The XRD patterns of the char residues correspond to TiO_2_ (PDF#21-1272/1278); and the Raman peaks of the char residues below 1000 cm^-1^ suggest that there is TiO_2_ phase in the char. These data suggest that TNTs are transformed into TiO_2_ during the combustion. Moreover, the *I*_D_/*I*_G_ (intensity ratio of D band to G band of GO) of GO-PVC is 1.6, and it declines to 1.1 for TNTs/GO-PVC, which suggests that the attendance of TNTs/GO can transform carbon sources into char.

**Reference**

[1] Li Q, Guo B, Yu J, et al. (2011) Highly efficient visible-light-driven photocatalytic hydrogen production of CdS-cluster-decorated graphene nanosheets, J. Am. Chem. Soc. 133: 10878.

[2] Williams G, Seger B, Kamat PV (2008) TiO_2_-graphene nanocomposites. UV-assisted photocatalytic reduction of graphene oxide, ACS Nano 2: 1487.

[3] Akhavan O, Ghaderi E (2009) Photocatalytic reduction of graphene oxide nanosheets on TiO_2_ thin film for photoinactivation of bacteria in solar light irradiation, J. Phys. Chem. C 113: 20214.

[4] Ryu B-Y, Emrick T (2011) Bisphenol-1,2,3-triazole (BPT) epoxies and cyanate esters: synthesis and self-catalyzed curing, Macromolecules 44: 5693.

[5] Zuiderduin WCJ, Westzaan C, Huetink J, Gaymans RJ (2003) Toughening of polypropylene with calcium carbonate particles, Polymer 44: 261.
